# Bortezomib-Induced Epigenetic Alterations in Nerve Cells: Focus on the Mechanisms Contributing to the Peripheral Neuropathy Development

**DOI:** 10.3390/ijms23052431

**Published:** 2022-02-23

**Authors:** Karolina Łuczkowska, Dorota Rogińska, Piotr Kulig, Anna Bielikowicz, Bartłomiej Baumert, Bogusław Machaliński

**Affiliations:** 1 Department of General Pathology, Pomeranian Medical University, 70-111 Szczecin, Poland; doroginska@gmail.com (D.R.); piotrkulig@interia.eu (P.K.); bielikowicz.anna@gmail.com (A.B.); 2 Department of Bone Marrow Transplantation, Pomeranian Medical University, 71-252 Szczecin, Poland; bartlomiej.baumert@pum.edu.pl

**Keywords:** bortezomib-induced peripheral neuropathy, multiple myeloma, bortezomib, dLUHMES

## Abstract

Bortezomib-induced peripheral neuropathy (BiPN) occurs in approximately 40% of patients with multiple myeloma. The induction of severe neuropathy entails the dose reduction or complete elimination of bortezomib (BTZ). Interestingly, discontinuation of BTZ mostly results in a reduction or complete resolution of peripheral neuropathy (PN) symptoms. Therefore, it is likely that the BiPN mechanisms are based on temporary/reversible changes such as epigenetic alterations. In this study, we examined the effect of treating nerve cells, differentiated from the Lund human mesencephalic (dLUHMES) cell line, with several low-dose BTZ (0.15 nM) applications. We showed a significant decrease in global histone H3 acetylation as well as histone H3 lysine 9 acetylation. Moreover, analysis of the genetic microarray showed changes mainly in epigenetic processes related to chromatin rearrangement, chromatin silencing, and gene silencing. GSEA analysis revealed three interesting signaling pathways (SIRT1, B-WICH and, b-Catenin) that may play a pivotal role in PN development. We also performed an analysis of the miRNA microarray which showed the interactions of miR-6810-5p with the genes *MSN, FOXM1, TSPAN9,* and *SLC1A5*, which are directly involved in neuroprotective processes, neuronal differentiation, and signal transduction. The study confirmed the existence of BTZ-induced complex epigenetic alterations in nerve cells. However, further studies are necessary to assess the reversibility of epigenetic changes and their potential impact on the induction/resolution of PN.

## 1. Introduction

Bortezomib (BTZ) is a potent chemotherapeutic drug widely used in the treatment of hematological malignancies, with particular emphasis on multiple myeloma (MM) and mantle cell lymphoma [1]. It acts as a selective and reversible 26S proteasome inhibitor, a crucial molecule in regulating the intracellular protein degradation pathway [2]. The drug attaches to the chymotryptic site and therefore inhibits the degradation of proteins that are vital for cell proliferation and survival. The blockage of these molecular pathways ultimately leads to apoptosis and growth inhibition [2]. BTZ can be administered either intravenously or subcutaneously, and a standard dose is 1.3 mg/m^2^ [3]. BTZ is rapidly cleared from plasma and distributed into the cellular compartment. The primary mechanism of inactivation occurs in the liver through intracellular oxidative deboronation via cytochrome P450 enzymes 3A4, 2D6, 2C19, 2C9, and 1A2 (27a). The terminal elimination half-life is estimated to be greater than 10 h and is dependent on dose and treatment cycle [2]. Although BTZ is being used predominantly in the treatment of hematological neoplasms, it was also under investigation in several solid tumors. Li et al. demonstrated that proteasome inhibitors can be potential therapeutics in the treatment of non-small cell lung carcinoma through downregulating c-Met and triggering cell death [4]. In another study, breast cancer cell lineages were exposed to BTZ alone and BTZ combined with IKKβ inhibitor BMS-345541. The obtained results depicted that the combination of IKKβ inhibitor and BTZ augmented the cytotoxicity of the latter [5]. Wu and colleagues conducted a study in which they investigated the anticancer effect of combined lower-dose irradiation and BTZ treatment on human oral cancer cells both in vitro and in a xenograft murine model. Obtained results suggest that BTZ enhances radiosensitivity through the suppression of radiation-induced TRAF6-NF-κB signaling activation [6]. Despite the proven efficacy in the treatment of hematological malignancies and satisfactory clinical outcomes, a good preclinical framework for further research in its application in different cancers, several adverse effects were reported. Peripheral neuropathy (PN) is one of the most common treatment-reducing, non-haematological side effects of BTZ treatment of MM, which often requires dose modification, delay, or drug discontinuation. [7]. It clinically manifests itself as neuropathic pain, located mainly distally, distal sensory loss to all modalities, suppression of deep tendon reflexes, and changes in proprioception. Pain and other sensory symptoms tend to be distributed in a sock and glove pattern [8]. The incidence of bortezomib-induced peripheral neuropathy (BiPN) is approximately 40% [9]. The exact etiology of BiPN is not entirely elucidated, but it is presumed to be multifactorial. Since the condition is at least partially reversible [9], one may presume that epigenetic mechanisms might be involved. ‘Epigenetics’ refers to changes in gene expression unrelated to changes to the DNA sequence itself, and is potentially reversible. Epigenetic marks include chromatin modifications (e.g., histone protein acetylation, methylation, and ubiquitination), noncoding long and small RNA (e.g., lncRNA, microRNA [miRNA], and PIWI-interacting RNA [piRNA]), and alterations to the DNA itself (e.g., DNA methylation and hydroxymethylation) [10]. Guo et al. examined DNA methylation profiling in patients with diabetic peripheral neuropathy (DPN). The results of their study suggest that DNA methylation is altered in sural nerves from subjects with DPN, and that the state of methylation may contribute to the regenerative ability of the nerve [11]. Moreover, PN in breast cancer patients treated with paclitaxel was associated with alterations in expression and methylation of genes of the hypoxia-inducible factor 1 signaling pathway [12]. The aforementioned studies suggest that epigenetic changes are involved in neurodegeneration, including drug-induced PN. Therefore, the contribution of epigenetic changes to the pathology of BiPN might at least partially explain its reversibility [9].

We also studied the phenomenon of BiPN. In one study, we examined 120 patients with MM treated with BTZ. The obtained results highlighted the importance of the role of proinflammatory cytokines. Moreover, we demonstrated the previously unknown effect of complement components on the development of BiPN [13]. In another study, we depicted that the exposure of PC12-derived nerve cells to BTZ leads to alterations in the miRNA expression in genes responsible for cell cycle, DNA repair, nerve processes and cell division. Disorders of these processes could have a negative influence on peripheral nervous system homeostasis, and may be associated with the neurotoxicity of BTZ [14].

Suspecting epigenetic alterations as the origin of PN, in the present study, we examined the effect of treating nerve cells, differentiated from the Lund human mesencephalic (dLUHMES) cell line, with several low-dose BTZ (0.15 nM) applications. We focused on epigenetic changes, with particular emphasis on alterations in miRNA expression and histone acetylation.

## 2. Results

### 2.1. Cell Viability

Nerve cell viability assessed 24 h after the fourth treatment with BTZ (0.15 nM) showed a statistically significant (*p* = 0.044) 3.67% increase in dead/apoptotic cells relative to control cells (ratio of red fluorescence to green fluorescence of JC-1 dye: control cells 7.71 ± 0.22; BTZ-treated cells 7.42 ± 0.30). The obtained results confirm the correctly selected dose for the experiment.

### 2.2. Gene Expression Profile in Neuronal Cells

**Figure 1:**
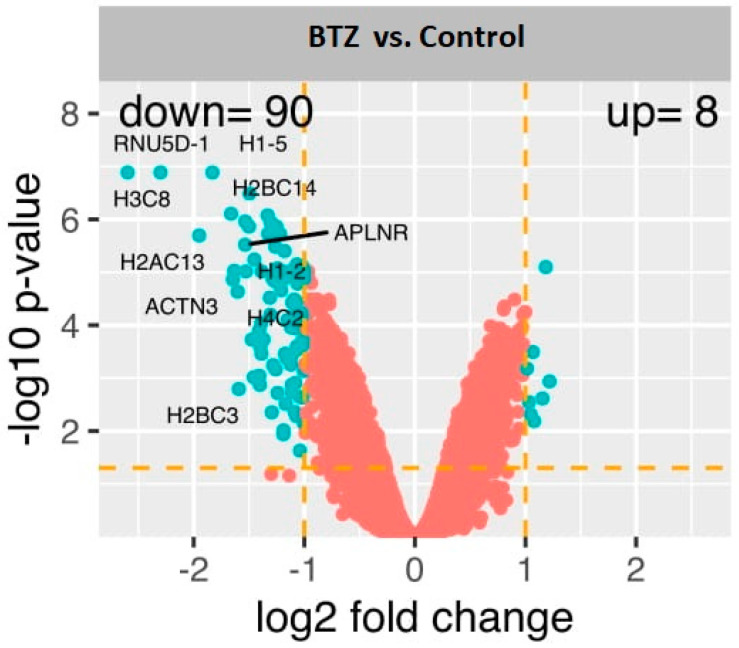
The volcano plot of global gene expression. Genes are represented by dots (*p* < 0.05, fold change > 2). The diagram also shows the symbol for the genes with the greatest expression change.

Genetic microarrays were performed in three technical repetitions for both control and BTZ-treated cells. Bioinformatics analysis of the gene expression showed 90 genes with reduced (fold −2 to −6.05; *p* < 0.05) and 8 genes with increased expression (fold 2 to 2.32; *p* < 0.05) in BTZ-treated cells compared to control cells (Figure 1).

**Figure 2:**
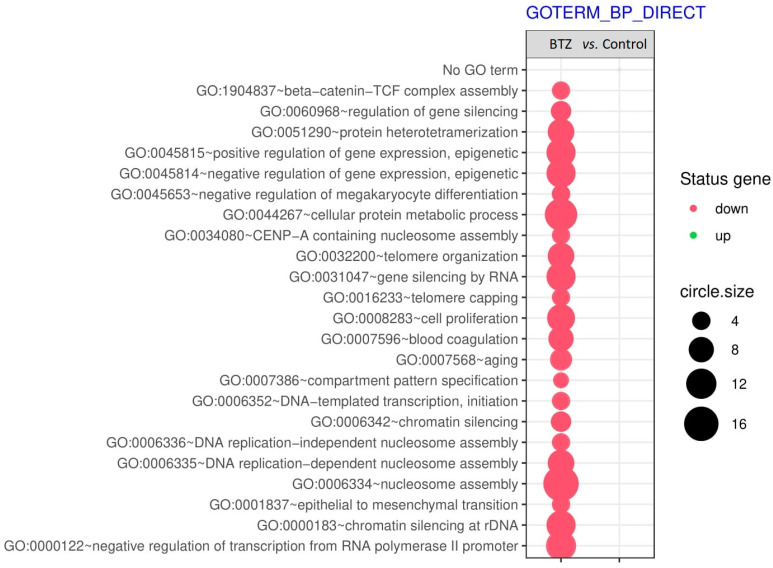
Bubble plot of biological processes according to GO classification.

Genes with significantly altered expression have been classified by gene ontology (GO) and assigned to the appropriate biological processes that they regulate (Figure 2). The results of this analysis are presented using a bubble plot with the following criteria: adj. *p* < 0.05; the Benjamini method used; for each process, there were a minimum of five genes—the size of the bubble corresponds to the number of genes involved in a given process, and the red color represents a reduced gene expression. The most important observed changes, significant for the development of PN, were mainly processes related to epigenetic changes, i.e., GO:0045815~positive regulation of gene expression, epigenetic; GO:0045814~negative regulation of gene expression, epigenetic; GO:0060968~regulation of gene silencing; GO:0031047~gene silencing by RNA; GO:0008283~cell proliferation; GO:0006342~chromatin silencing; GO:0006336~DNA replication−independent nucleosome assembly; GO:0000183~chromatin silencing at rDNA.

**Figure 3:**
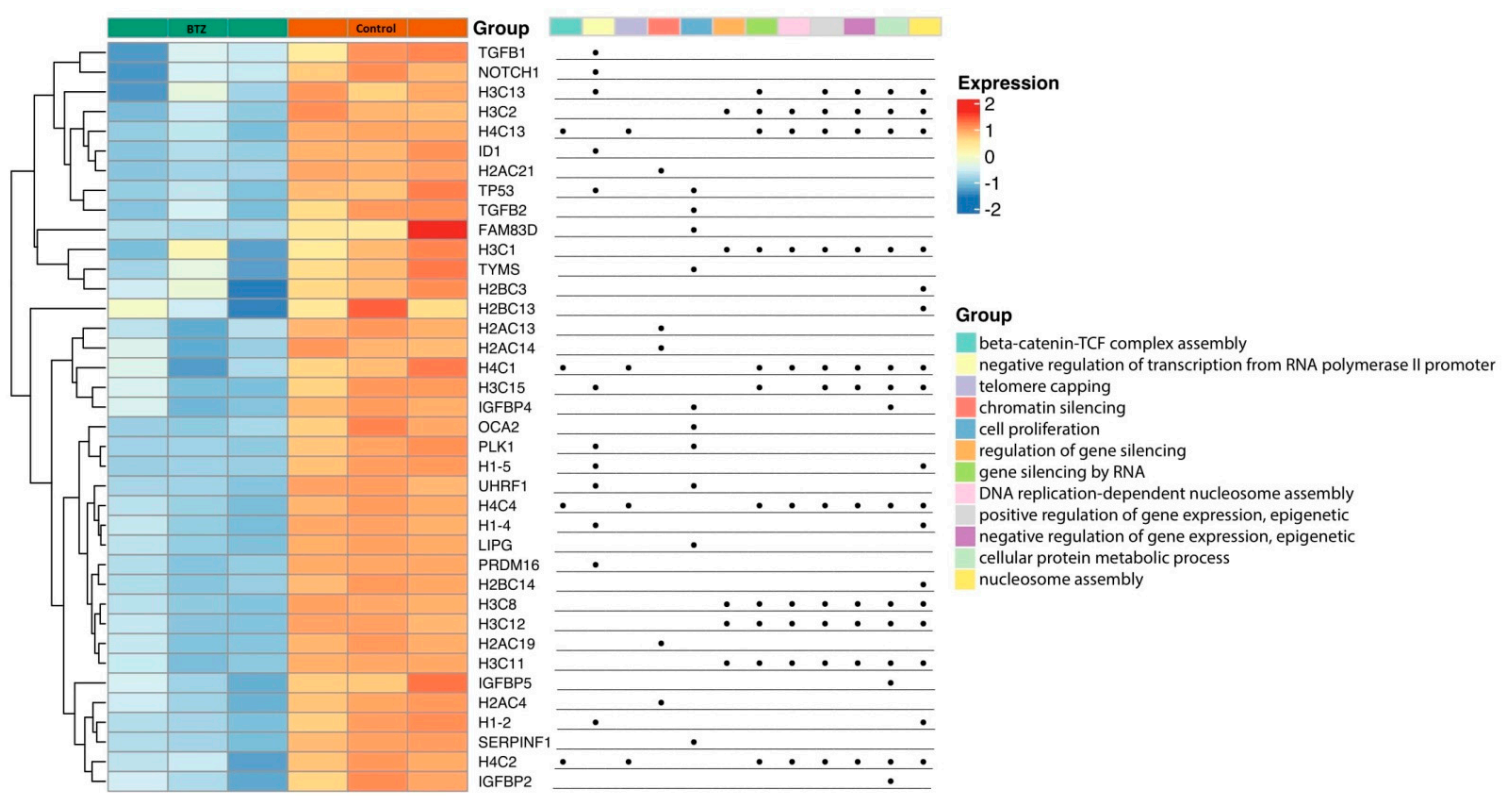
Expression of selected genes presented as a heatmap. Red color means increased gene expression and blue decreased gene expression. In addition, specific genes have been assigned to regulate biological processes (the dot indicates the gene’s participation in a specific process).

Then, based on the results from the bubble plot, the processes with the largest number of genes were thoroughly analyzed. Details are shown in Figure 3. This figure includes both a global view and a detailed look at specific genes whose expression was reduced by BTZ treatment (*p* < 0.05). Moreover, these genes have been assigned to the particular processes that they regulate.

#### Gene Set Enrichment Analysis

**Figure 4:**
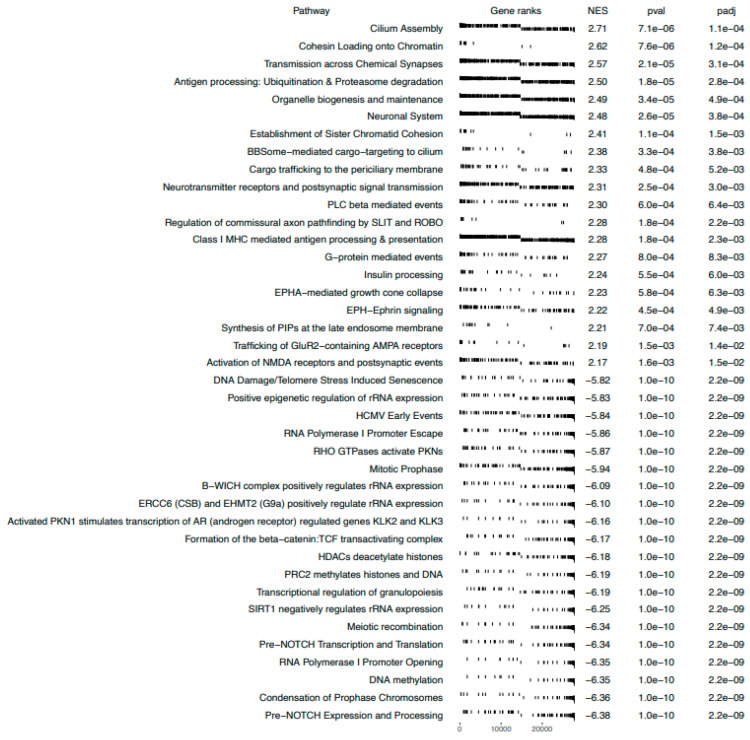
GSEA in BTZ-treated dLUHMES cells. Gene sets are ranked according to the normalized enrichment score (NES).

Gene set enrichment analysis (GSEA) uses sets of a priori genes that have been grouped according to their participation in the same biological pathway. GSEA puts emphasis on the analysis of gene groups and not on the operation of individual genes, distinguishing a specific phenotype. GSEA identified, in BTZ-treated cells, 20 sets of genes with a positive correlation and 20 sets of genes with a negative correlation (Figure 4). The following processes seem to be the most relevant: ERCC6 (CSB) and EHMT2 (G9a) positively regulate rRNA expression; the B−WICH complex positively regulates rRNA expression; SIRT1 negatively regulates rRNA expression; DNA damage/telomere stress-induced senescence; and DNA methylation. In addition, the first two processes directly affect histone H3 acetylation, which we tested in this study using the ELISA and WB methods.

### 2.3. Validation of mRNA Microarray

For the validation of the microarray, we selected seven genes (with the highest fold changes) essential for the functioning of the nervous system. The validation was performed using the qRT-PCR method. Analysis of the expression of selected genes using the qRT-PCR method confirmed the results obtained from the genetic microarray. Figure S1 shows the expression of individual genes from both qRT-PCR (A) and microarrays (B) (Supplementary Materials).

### 2.4. miRNAs Expression Profile in Neuronal Cells

**Figure 5:**
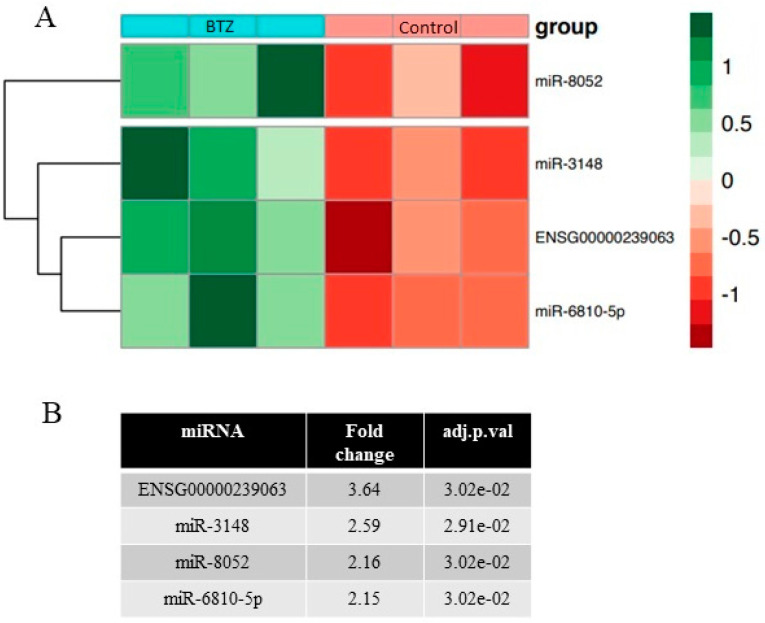
(**A**) Expression of selected miRNAs presented as a heatmap. Red color means decreased miRNA expression and green increased miRNA expression. (**B**) Fold change for miRNAs in BTZ-treated vs. control cells.

miRNA microarrays were performed in three technical repetitions for both control and BTZ-treated cells. Bioinformatics analysis of the miRNA expression showed 0 miRNAs with reduced and 4 miRNAs with increased expression (fold 2.15 to 3.64; *p* < 0.05) in BTZ-treated cells compared to control cells. Detailed data on changes in miRNA are presented on the heatmap (Figure 5).

**Figure 6:**
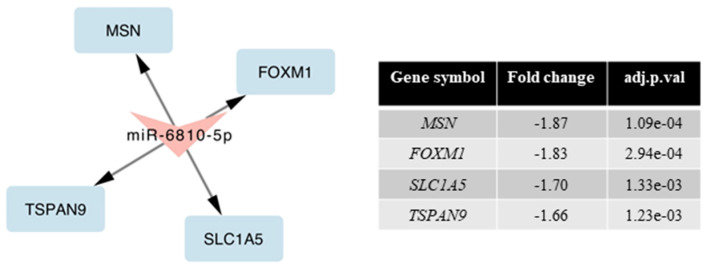
mRNA–miRNA interactions (BTZ-treated vs. control cells).

Moreover, we analyzed the mRNA–miRNA interaction. We compared the results obtained with the mRNA arrays to the miRNA arrays. We observed four genes with reduced expression (Figure 6), whose expression is regulated by miRNA-6810-5p. Consequently, an increase in miR-6810-5p expression may inhibit the expression of its target genes. According to the miRDB v. 6.0 database, miR-6810-5p has 481 target genes with a score in the range of 50–98. Seventy-two target genes have target score > 80, which means high confidence targets. The two genes identified by us have a very high target score (*FOXM1* = 91; *SLC1A5* = 96). The identified genes play a significant role in maintaining the homeostasis of the nervous system (41–45).

### 2.5. Validation of miRNA Microarray

For the validation of the miRNA microarray, we selected three miRNAs (with the highest fold changes) essential for the functioning of the nervous system. The validation was performed using the qRT-PCR method. Analysis of the expression of selected miRNAs using the qRT-PCR method confirmed the results obtained from the genetic microarray. Figure S2 shows the expression of individual miRNAs from both qRT-PCR (A) and microarrays (B) (Figure S2).

### 2.6. Histone Acetylation

#### 2.6.1. ELISA

**Figure 7:**
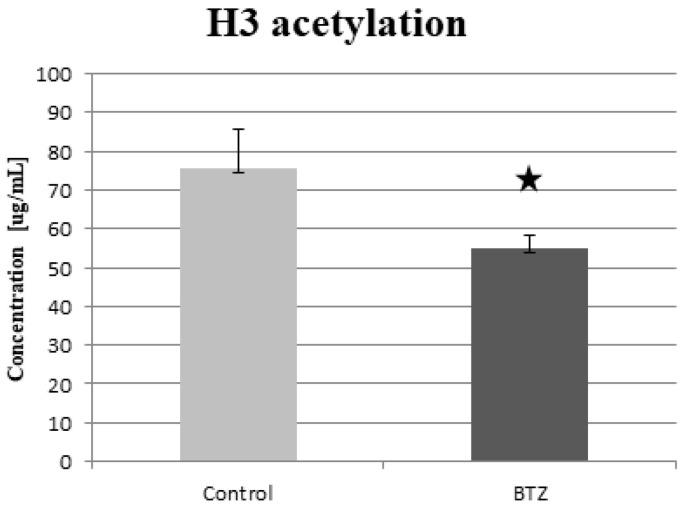
The concentration of global acetylated histone H3 in dLUHMES cells treated with BTZ and in the control cells. * *p* < 0.05.

The measurement of global histone H3 acetylation by the ELISA method showed a statistically significant (*p* = 0.026) decrease in the level of acetylation in BTZ-treated cells. A reduced 27.16% level of acetylation was observed in BTZ-treated cells compared to control cells (Figure 7). Simultaneously, the measurement of global histone H4 acetylation showed no difference in BTZ-treated cells compared to control cells (data not shown).

#### 2.6.2. Western Blot

**Figure 8:**
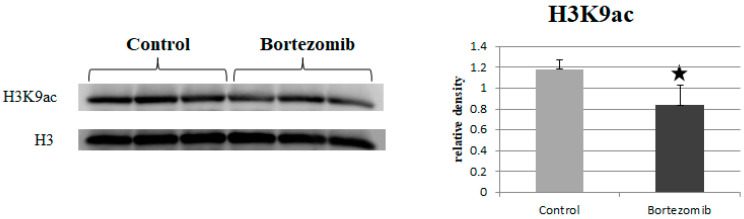
Western blot quantification of H3K9 acetylation in dLUHMES cells treated with BTZ and in the control cells. Data are presented as mean ± SD (*n* = 3). * *p* < 0.05. H3K9ac and H3 bands observed at 17 kDa.

The results obtained by the Western blot method showed a statistically significant (*p* = 0.041) decreased level of histone H3K9 acetylation in dLUHMES cells treated with BTZ vs. control cells (Figure 8). The remaining H3 and H4 acetylation sites tested (H3K4ac, H3K14ac, H4K8ac, H4K5ac, H4K16ac) showed no significant differences between the groups (data not shown).

## 3. Discussion

The problem of drug-induced neuropathy in patients treated with BTZ is common and often prevents proper therapy. The mechanisms of neuropathy development are not fully elucidated due to its heterogeneous nature. However, epigenetic changes appear to be crucial in the development of this process. They can temporarily alter gene expression by disrupting the homeostasis of the nervous system. Epigenetic changes include histone and DNA modifications such as acetylation, methylation, and phosphorylation, and changes in gene expression as a result of the action of miRNA molecules. All epigenetic modifications can be temporary and change throughout life. They have been shown to be responsible for the development of many diseases, such as cancers, neurodegeneration, or cardiovascular diseases [15,16,17]. In the case of BiPN, in some patients with MM after the dose reduction or discontinuation of BTZ, symptoms decrease or completely disappear [17,18,19]. The severity of neuropathy and the time it occurs during BTZ treatment are individual to the patient. BiPN most often appears in the first cycles of treatment, reaching its plateau in the 5th cycle [19]. Symptoms may appear as early as the first cycle of chemotherapy, but usually require more than one dose [20]. BTZ is administered to patients as a short intravenous infusion or by the subcutaneous route on certain treatment days (days 1, 4, 8, 11 in a 3-week cycle) at a therapeutic dose of 1.3 mg/m2 [21]. In clinical trials, the highest degree of inhibition of the 20S proteasome was observed within the first hour after the administration of BTZ [22]. Typically, the elimination time is between 40 and 193 h. It is noteworthy that after the first dose of the drug, its t_1/2_ is about 13 h, while after 11 days of treatment in the same regimen, t_1/2_ is prolonged and amounts to about 76 h [23]. Therefore, in our study, we used a quadruple treatment of the nerve cells at the same intervals as for the treatment of the patient to mimic the clinical conditions as much as possible.

Genetic microarrays mainly showed changes in the processes involved in epigenetic modifications, (Figure 2) such as the regulation of gene expression–epigenetic, regulation of gene silencing, chromatin silencing, and DNA replication–independent nucleosome assembly. BTZ is able to initiate epigenetic changes by altering the level of global DNA methylation in MM patients [24] and is involved in the regulation of miRNAs’ levels [14,25,26]. In our previous studies [27], we have shown significant epigenetic changes, manifested by a complete change in the DNA methylation profile, induced by the action of BTZ on cancer neuroblastoma cells. We have also observed that DNA methylation changes transferred to daughter cells are possibly involved in the development of resistance to BTZ. In this study, GSEA bioinformatics analysis (Figure 4) revealed interesting metabolic pathways that may be directly involved in the development of BiPN. One of them was the sirtuin 1 (SIRT1) signaling pathway. The protein SIRT1 has a neuroprotective and neuroregenerative effect [28] and influences the differentiation of neurons [29]. This factor stimulates mTOR-independent autophagy, improving motor axon regeneration [30]. Moreover, its neuroregenerative effect was shown in a rat model in retrograde degeneration in motor neurons in the spinal cord of rats after peripheral nerve root injury [28].

Chen et al. demonstrated the effect of BTZ on the expression of SIRT1 and the development of neuropathy in a rat model. BTZ was administered to rats intraperitoneally at a dose of 0.4 mg/kg daily for 5 days to induce polyneuropathy. Moreover, some rats received SITR1 activator (resveratrol) concurrently. The study detected a decreased expression of SIRT1 in the L4-L6 spinal dorsal horn in rats treated with BTZ, while in rats treated with BTZ and SITR1 activator resveratrol, an increase in SIRT1 expression and a significant reduction in the symptoms of neuropathy was observed [31]. Therefore, reducing the expression of genes that regulate this signaling pathway may have a meaningful impact on the appearance of PN symptoms.

Then we observed a decreased level of the genes involved in the B-WITCH signal path. The B-WICH complex is responsible for maintaining the homeostasis of the nervous system through chromatin remodeling. As a result of the reduction of the expression of proteins (WSTF, SNF2h, NM1) included in the B-WITCH complex, the expression of 45S pre-rRNA is reduced and the chromatin structure is condensed. As a consequence, the level of histone H3 acetylation, in particular H3K9, is reduced [32]. A similar mechanism is also controlled by the ERCC6 (CSB) pathway, the reduced expression of which was demonstrated by GSEA analysis. ERCC6 is responsible for the induction of histone H3K9 acetylation by recruiting and binding PCAF to rDNA. Knockdownof *PCAF* or *ERCC6* has been shown to reduce the level of H3K9ac in rDNA promoters, prevent RNA polymerase I association and inhibit pre-rRNA synthesis [33]. In our study, we confirmed a reduced level of global acetylation of H3 (Figure 7) and H3K9 (Figure 8), which may be a direct result of the silencing of the B-WITCH or ERCC6 signal path. Another signaling pathway important for the proper functioning of the nervous system is b-Catenin. In our study, we observed a statistically significant reduction in this signaling pathway in the GSEA analysis. b-Catenin regulates the self-renewal of neural progenitor cells and the differentiation of neurons [34]. BTZ has been shown to reduce the expression of the Wnt/β-catenin signaling pathway in RPMI-8226 myeloma cells. The decrease in the expression of this signaling pathway was probably associated with a decrease in proliferation and an increase in apoptosis in neoplastic cells [35]. Similar results were obtained in an in vivo experiment in a mouse prostate cancer model. The administration of BTZ slowed down tumor growth and increased survival. qRT-PCR and Western blots showed an increase in the expression of antiproliferative genes and an inhibition of the Wnt/β-catenin signaling pathway [36].

As was mentioned above, we observed a decrease in the acetylation of H3 and H3K9 histones. According to the available literature, BTZ reduces acetylation of H3 histones; however, some reports have obtained opposite results. BTZ treatment of breast cancer cells (MCF7 cells) showed a significant reduction in histone H3 acetylation and histone acetyltransferase p300 [37]. On the other hand, in another cancer cell line, BTZ treatment induced an increase in H3 acetylation [38]. Therefore, the differences in the effect of BTZ on cells are probably due to the type of cells, the dose of BTZ used, the amount of BTZ exposure during the test, and the exposure time. Our research showed a decrease in the global acetylation of H3 and H3K9 after treating nerve cells 4 times with BTZ (0.15 nM). Additionally, the obtained results correlate with the signaling pathways that are responsible for the process of histone acetylation (ERCC6 or B-WITCH) demonstrated in microarrays. We did not observe significant changes in the expression of genes directly involved in acetylation (*HAT1, KAT*) or deacetylation (*HDAC*) of histones in the genetic microarray. In the previous study [14], after a single treatment with BTZ of the PC12 neural cell line, we observed a simultaneous reduction of genes responsible for histone acetylation *HAT1* (fold = −2.9), *ESCO2* (fold = −8.83) and deacetylation *HDAC5* (fold = −2.95), *HCAA2* (fold = −4.92). However, we did not check global histone acetylation by ELISA or Western blot.

Regulation of gene expression by miRNA is one type of epigenetic change. miRNAs regulate the expression of thousands of genes affecting the functioning of the body. BTZ influences the expression of individual miRNAs that regulate the expression of their target genes. Duan et al. have shown in a rat model the association of blocking miR-155 expression with the development of BiPN. Blocking miR-155 expression reduced mechanical allodynia and thermal hyperalgesia in rats [39]. In our previous study [40], we showed the relationship between changes in plasma miRNA expression (miR-22-3p; miR-23a-3p; miR-24-3p) in patients with MM with the development of drug-induced peripheral neuropathy. These studies confirmed that the treatment affects epigenetic modification and may be related to the development of neuropathy.

In this study, we performed a global analysis of miRNAs’ expression in nerve cells after exposure to BTZ. In addition, we analyzed the correlation of the results obtained from the analysis of the mRNA and miRNA genetic microarrays. We associated the observed changes in miRNA expression with target genes that were dysregulated. It is a method that allows the actual changes resulting from the mechanism of action of miRNA on target genes to be shown. One of the interesting observations was a significant increase in the expression of miRNA-6810-5p (fold = 2.15), which decreased the expression of four target genes closely related to the nervous system (*FOXM1, SLC1A5, TSPAN9,* and *MSC*). The significant role of the *FOXM1* gene in the control of the length of the cell cycle of neuronal precursors and the regulation of their fate after the division has been proven, by knocking down the *FOXM1* gene, which resulted in a reduction in the regeneration of the spinal cord after an injury [41]. In studies carried out on cerebellar neural stem cells, it was shown that *FOXM1* regulates the expression of miRNA-miR-130b, miR-301a, miR-15~16, and miR-17~92, the knockdown of which significantly impaired the ability to create the neurosphere [42]. Moreover, the *FOXM1* gene is the main target gene of proteasome inhibitors (including BTZ). Drugs from this group reduce the expression of *FOXM1*, thus limiting the proliferation of neoplastic cells [43], but its increased expression was observed in myeloma cells in relapsed patients, which may indicate the involvement of this gene in the development of resistance to BTZ [44]. The *SLC1A5* gene encodes a membrane transporter responsible for the intracellular transport of amino acids. This gene shows high expression in cells of the nervous system, ensuring their proper functioning [45].

Epigenetic changes that arise because of the action of BTZ cause global changes in the functioning of cells. As we showed in our previous study [27], treatment of SH-SY5Y tumor cells with a single dose of BTZ caused a complete change in the DNA methylation profile, which is probably responsible for the development of resistance to BTZ. This proves the multidirectional activity of BTZ and its influence on the dysregulation of thousands of genes and miRNA molecules, disrupting many signaling pathways and biological processes. Therefore, the development of BiPN is a complex process that cannot be considered in one way. More and more scientific reports confirm that epigenetic changes are the basis for disturbances in gene expression and signaling pathways in nerve cells. Perhaps counteracting the emerging epigenetic change in nerve cells would stop the entire cascade of dysregulation and reduce/eliminate the development of polyneuropathy.

This study confirmed the existence of bortezomib-induced complex epigenetic alterations in nerve cells. However, further in-depth studies in MM patients are necessary to correlate the detected epigenetic changes with the potential presence of PN. The next step will be to assess the reversibility of epigenetic changes and their potential impact on the induction/resolution of PN.

### Study Limitations

Despite our best efforts and the use of the latest molecular biology tools, this study has some drawbacks. The neuronal cell line we used (dLUHMES), although great for studying the mechanisms of neurodegenerative diseases and epigenetic alterations in neurons, has one limiting constraint. Namely, the maximum cell culture time for dLUHMES is 21 days. This means that it is not possible to use this line to assess epigenetic changes, e.g., after 2, 3, or 4 cycles of BTZ therapy, thus mimicking typical clinical conditions. However, according to the available literature [46,47,48,49,50,51], this problem would likely also occur in other non-proliferating neuronal lines with a standard cell culture time of 21 days. Simultaneously, for the same reasons, it is not possible to reliably assess the reversibility of the detected epigenetic changes after the conducted experiment. In the future, we plan to use a multi-drug model to further reflect the development of neuropathy during the treatment of patients with MM. Nevertheless, the performed studies mark a new direction for further research on the development of BiPN. The obtained research should be successively confirmed on an animal model and then referred to clinical trials.

## 4. Materials and Methods

### 4.1. Cell Culture and Differentiation

Lund human mesencephalic (LUHMES) cells, i.e., human neuronal precursor cells were applied in this study. According to the literature, differentiated LUHMES (dLUHMES) cells show favorable features for neurotoxicity studies because they differentiate relatively quickly, have a higher level of expression of neuronal markers than other neuronal cell lines such as SH-SY5Y or neural stem cells (NSCs), and are more sensitive to a number of neurotoxins [50]. LUHMES cells were incubated for growth in T-75 flasks coated by fibronectin (1 mg/mL) (Sigma-Aldrich, Darmstadt, Germany) and poly-L-ornithine (1 mg/mL) (Sigma-Aldrich, Darmstadt, Germany) in Advanced DMEM/F-12 medium (ThermoFisher Scientific, Waltham, MA, USA), medium modified to contain N-2 supplement (100x) (ThermoFisher Scientific, Waltham, MA, USA), L-Glutamine (1 mg/mL) (Sigma-Aldrich, Darmstadt, Germany), human basic fibroblast growth factor (160 µg/mL) (bFGF, R&D Systems, Minneapolis, MN, USA), and penicillin (100 U/mL) (Sigma-Aldrich, Darmstadt, Germany) at 37 °C in a saturated humidity atmosphere containing 5% CO_2_. The proliferation medium was changed every 2 days. LUHMES cell differentiation into neurons was conducted according to the protocol described by Harris et al. [51]. LUHMES cell differentiation medium contained: advanced DMEM/F-12 medium, N-2 supplement (100x), L-Glutamine (1 mg/mL), cAMP (100 mM) (R&D Systems, Minneapolis, MN, USA), Tetracycline (2 mg/mL) (Sigma-Aldrich, Darmstadt, Germany), and glial cell line-derived neurotrophic factor (20 μg/mL) (GDNF, R&D Systems, Minneapolis, MN, USA). The differentiation lasted for 7 days.

### 4.2. The Course of the Experiment and the Determination of the BTZ Dose

dLUHMES cells were not treated (control) or treated 4 times with BTZ 0.15 nM (Cell Signalling Technology, Danvers, MA, USA). Each time, the cells were incubated with BTZ medium for 24 h. The medium was changed to BTZ-free medium between each treatment (Table 1). The experimental schedule was designed to closely resemble the treatment regimen of MM patients. The standard treatment regimen in MM patients with BTZ is to administer the drug 4 times (on day 1, 4, 8, 11) in each cycle. Then a 10 day break is required and another treatment cycle begins [17,18]. In the original version of the experiment, we performed cell treatments simulating two treatment cycles. However, the time of cell culture turned out to be too long, and we observed in the results of genetic microarray a significant increase of apoptotic/death cell processes in control cells, probably related to the aging of the cell culture. According to the protocol [51], the maximum cell culture time for dLUHMES is 21 days. In summary, the cell differentiation process and the experiment with 2 treatment cycles lasted 40 days (7 days of differentiation, 11 days of the first cycle, 10 days break, and 12 days of the next cycle). Therefore, in this article, we present the results only after 1 treatment cycle (7 days of differentiation, 12 days of the first cycle).

The aim of this experiment was to demonstrate changes at the molecular level that may lead to the development of BiPN. Most often, PN in patients occurs after the repeated administration of BTZ, but reducing the dose, changing the route of administration, or changing the treatment regimen results in a reduction or complete elimination of symptoms. Therefore, the changes leading to the development of PN probably do not result from the death of neurons, but only from a temporary disturbance of their functioning, e.g., by epigenetic modifications. Therefore, in order to notice significant changes at the molecular level, low multiple doses of BTZ were used, which only slightly reduced cell viability (2.1. Cell viability). In our previous study [14], we applied a high (50 nM) single dose of BTZ to nerve cells, and the obtained results mainly included increased apoptosis and neuronal death.

### 4.3. RNA Isolation

Total RNA was isolated from three separate cell incubations for the control group as well as the BTZ group. 1.5 × 10^6^ dLUHMES cells were used each time to isolate one RNA sample. The applied isolation kit (mirVana™miRNA Isolation Kit, ThermoFisher, Waltham, MA, USA) allows for the simultaneous isolation of total RNA enriched with miRNA molecules. RNA isolation was performed according to the manufacturer’s protocol. Genetic material was always eluted from the column at 30 µL. The concentration and quality of the isolated RNA was measured with an Epoch spectrophotometer (Biotek, Winooski, VT, USA).

### 4.4. Histone Isolation

Histones were isolated from three separate cell incubations for the control group as well as the BTZ group. 1.5 × 10^6^ dLUHMES cells were used each time to isolate one histone sample. The Histone Extraction Kit (Abcam, Cambridge, UK) was used to isolate the histones. Histone isolation was performed according to the manufacturer’s protocol. The obtained histone concentration was measured by the Bradford method using the Varioskan LUX (ThermoFisher, Waltham, MA, USA).

### 4.5. Cell Viability

Twenty-four hours after the fourth treatment with BTZ (0.15 nM), cell viability was examined by measuring the mitochondrial membrane potential (ΔψM) (JC-1 Mitochondrial Membrane Potential Assay Kit, Cayman, Ann Arbor, MI, USA). JC-1 is a lipophilic, cationic dye whose color changes as the membrane potential increases. In healthy cells, a high level of mitochondrial potential (ΔψM) is observed, therefore J aggregates (JC-1) forms with intense red fluorescence. In contrast, in apoptotic/unhealthy cells, there is a low (ΔψM) and JC-1 remains in a monomeric form which shows green fluorescence. Cell staining after four times incubation with BTZ was performed according to the manufacturer’s protocol in a 96-well plate. Staining of 8 wells with control cells and 8 wells with BTZ cells was performed. Each well contained 5 × 104 cells. Fluorescence was measured at 30 points in each well at two wavelengths (Ex535 nm/Em595 nm; Ex485 nm/Em535 nm). The ratio of red fluorescence intensity to green fluorescence intensity was used as an indicator of cell health. Fluorescence was measured using Varioskan LUX (ThermoFisher, Waltham, MA, USA).

### 4.6. Affymetrix GeneChip Microarray and Data Analysis

The Ambion WT Expression Kit (Thermo Fisher Scientific, Waltham, MA, USA) was used to generate a cDNA strand that was fragmented using the GeneChip WT Terminal Labeling Kit (Affymetrix, Santa Clara, CA, USA). The obtained fragments were then hybridized onto an Affymetrix Human Gene 2.1 ST Array Strip. Subsequent fluidization and scanning steps were performed with the Affymetrix GeneAtlas System, with designated software (Affymetrix, Santa Clara, CA, USA). Bioinformatics analysis was performed in the statistical programming language R using BioConductor software. The robust multiarray average (RMA) normalization algorithm in the “Affy” library was used for the normalization, background correction, and calculation of the expression level of all tested genes. A detailed description of the mRNA microarray was described in our previous articles [14,26].

### 4.7. Affymetrix GeneChip miRNA Microarray

miRNA arrays were made using an miRNA 4.1 Array Strip Human (Affymetrix, Santa Clara, CA, USA). Affymetrix GeneAtlas system was used to scan the miRNA arrays. ll bioinformatics analyzes were performed using BioConductor. A detailed description of the miRNA microarray analysis and miRNA–RNA correlation was described in our previous articles [14,26].

### 4.8. Validation of Data Obtained from Microarrays

#### 4.8.1. Validation of mRNA Microarray

The qRT-PCR reaction was performed using a Bio-Rad CFX96 Real-Time PCR Detection System (Bio-Rad Inc., Hercules, CA, USA). Primers were designed using the BLAST program and then purchased from the Laboratory of DNA Sequencing and Oligonucleotide Synthesis of the Institute of Biochemistry and Biophysics of the Polish Academy of Sciences in Warsaw. First, the isolated total RNA (0.1 µg/sample) from dLUHMES cells was reverse transcribed using the First Strand cDNA synthesis kit (Thermo Fisher Scientific, Waltham, MA, USA) according to the manufacturer’s protocol. Then, the cDNA was used to analyze the expression of the selected genes. The qRT-PCR reaction mixture consisted of: 5 µL SYBR Green PCR Master Mix (Bio-Rad Inc., Hercules, CA, USA); 1 µL cDNA template; 2.8 μL nuclease-free water; and 0.6 µL of a specific forward primer and 0.6 of a specific reverse primer. BMG was used as an endogenous control gene. The relative expression of analyzed genes was calculated as 2 ^−ΔΔCT^.

#### 4.8.2. Validation of miRNA Microarray

The isolated miRNA (0.1 µg) was reverse transcribed using the qScript microRNA cDNA Synthesis Kit (Quanta Biosciences, Beverly, MA, USA) according to the manufacturer’s protocol. The qRT-PCR reaction mixture contained: 5 µL PerfeCTa SYBR Green SuperMix (Quanta Biosciences, Beverly, MA, USA); 0.2 µL microRNA specific primer; 0.2 µL PerfeCTa universal PCR primer (Quanta Biosciences, Beverly, MA, USA), 1 µL microRNA cDNA; 4.6 μL nuclease-free water. miR-93 was used as the endogenous control miRNA. The relative expression of the analyzed genes was calculated as 2 ^−ΔΔCT^.

### 4.9. Histone Acetylation

#### 4.9.1. ELISA

In this study, the global acetylation of histones H3 and H4 was measured. The following kits were used to measure histone acetylation: EpiQuik Total Histone H3 Acetylation Detection Fast Kit (Fluorometric); EpiQuik Total Histone H4 Acetylation Detection Fast Kit (Fluorometric) (EpiGentek, Farmingdale, NY, USA). Cell lysates were the material for measuring the acetylation level of selected histones by ELISA. The test was performed in *n* = 3 technical replicates for control as well as BTZ cells. The test was performed according to the manufacturer’s protocol. In brief, the first step was adding to the 96-well plate antibody buffer, cell lysates (5 ng/well), and standards (7 points: 1.5, 3, 6, 12, 25, 50, and 100 ng/μL). The plate was incubated for 1 h at room temperature (RT). Then the contents of the wells were rinsed three times and a detecting antibody was added. After an hour at RT on an orbital shaker (100 rpm) the wells were rinsed 6 times and fluoro-development solution was added for 5 min, and the fluorescence was measured at 530ex/590em nm using the Varioskan LUX (Thermo Fisher Scientific, Waltham, MA, USA).

#### 4.9.2. Western Blot

The results obtained from the analysis of the global histone H3 acetylation prompted us to continue looking for changes in the acetylation of specific lysines. Therefore, we decided to use the Western blot method due to the limited amount of histone extract. The ELISA method each time consumes large amounts of material, while the Western blot method allowed for the evaluation of 7 places on the H3 and H4 histones, using a much smaller amount of material compared to the ELISA method.

Initially, the histone samples were incubated for 5 min at 95 °C in a dry bath. Next, the histone samples (20 µg/well) and protein standards (Precision Plus Protein™ All Blue Prestained Protein Standards, Bio-Rad Inc., Hercules, CA, USA)were applied and separated (for 30 min, 200 V) on a 4–20% sodium dodecyl sulfate polyacrylamide gel electrophoresis (SDS-PAGE, miniPROTEAN II electrophoresis system, Bio-Rad Inc., Hercules, CA, USA) and next transferred to a 0.2 µm polyvinylidene fluoride (PVDF) membrane (Bio-Rad Inc., Hercules, CA, USA). The transfer was carried out for 70 min at 30 V. After 2 h blocking in 3% bovine serum albumin (BSA) at RT conditions, the membrane was probed with a specific primary antibody as follows: H3K9ac (at 1:1000 dilution) (ThermoFisher, Waltham, MA, USA, cat. number MA5-33384-20UG); H3K4ac (at 1:1000 dilution) (ThermoFisher, Waltham, MA, USA, cat. number MA5-24673); H3K14ac (at 1:1000 dilution) (ThermoFisher, Waltham, MA, USA, cat. number MA5-32814); H4K8ac (at 1:1000 dilution) (ThermoFisher, Waltham, MA, USA, cat. number 701796); H4K5ac (at 1:1000 dilution) (ThermoFisher, Waltham, MA, USA, cat. number MA5-32009); H4K16ac (at 1:1000 dilution) (ThermoFisher, Waltham, MA, USA, cat. number MA5-27794); Histone H3 (at 1:1000 dilution) (ThermoFisher, Waltham, MA, USA, cat. number MA5-15150). A secondary antibody (specific for the primary antibody used in the previous step) conjugated with horseradish peroxidase (HRP) was used to visualize the bands. Chemiluminescence detection was performed using the ECL Advance Detection Kit (Amersham Life Sciences, Buckinghamshire, UK), and the bands were visualized with a UVP camera (Gel DOC-It Imaging system, Bio-Rad Inc., Hercules, CA, USA). The ImageJ software ver. 1.8.0 (NIH, WI, USA) was used to determine the background-subtracted density of the bands. The relative histones acetylation levels were quantified in comparison to those of H3 and H4 histones.

### 4.10. Statistical Methods

The arithmetical means and standard deviations (SD) were calculated using MS Excel 2016. Comparisons of parameters between two groups were made using unpaired Student’s *t*-test and *p*-value < 0.05 was considered statistically significant.

## Figures and Tables

**Table 1:** Schedule of BTZ treatment of dLUHMES cells.

Day of the Experiment	Action
1st	medium with BTZ (0.15 nM) for 24 h
2nd	change the medium to BTZ-free
3rd	
4th	medium with BTZ (0.15 nM) for 24 h
5th	change the medium to BTZ-free
6th	
7th	change the medium to BTZ-free
8th	medium with BTZ (0.15 nM) for 24 h
9th	change the medium to BTZ-free
10th	
11th	medium with BTZ (0.15 nM) for 24 h
12th	RNA/miRNA and histone isolation

## Data Availability

The data presented in this study are available upon request from the corresponding author.

## Supplementary Materials

The following are available online at https://www.mdpi.com/article/10.3390/ijms23052431/s1.
