# Supplementary figures and images for: Bortezomib-Induced Epigenetic Alterations in Nerve Cells: Focus on the Mechanisms Contributing to the Peripheral Neuropathy Development
Source: Int J Mol Sci. 2022 Feb 23;23(5):2431. doi: 10.3390/ijms23052431 (PMC8910765; doi:10.3390/ijms23052431)

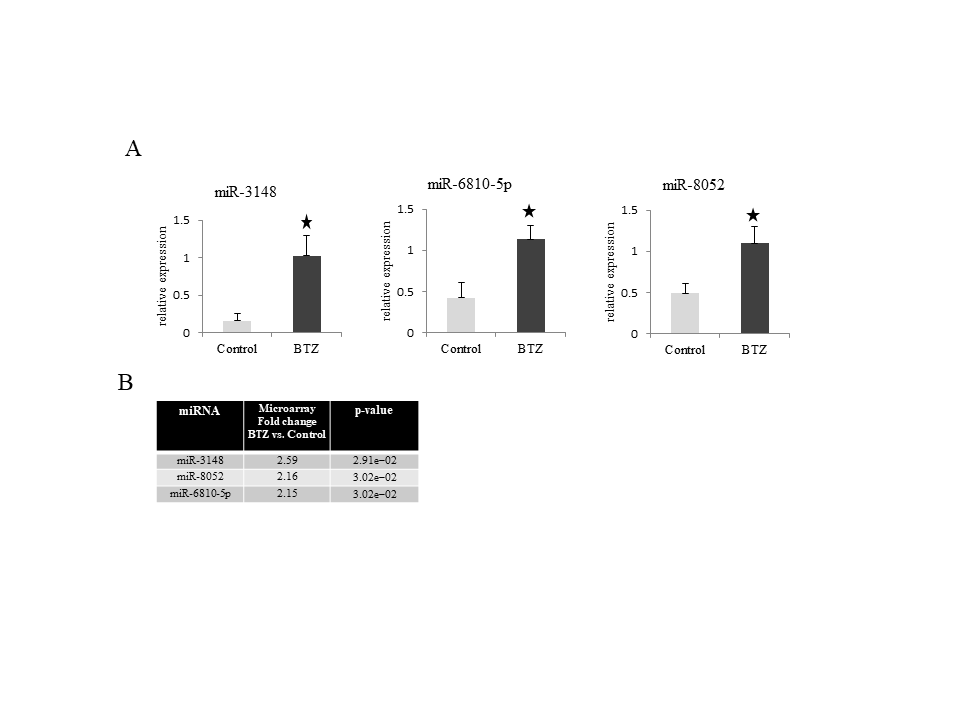

Supplement: Supplementary file 1 [file ijms-23-02431-s001.zip › fig. S2.tif]

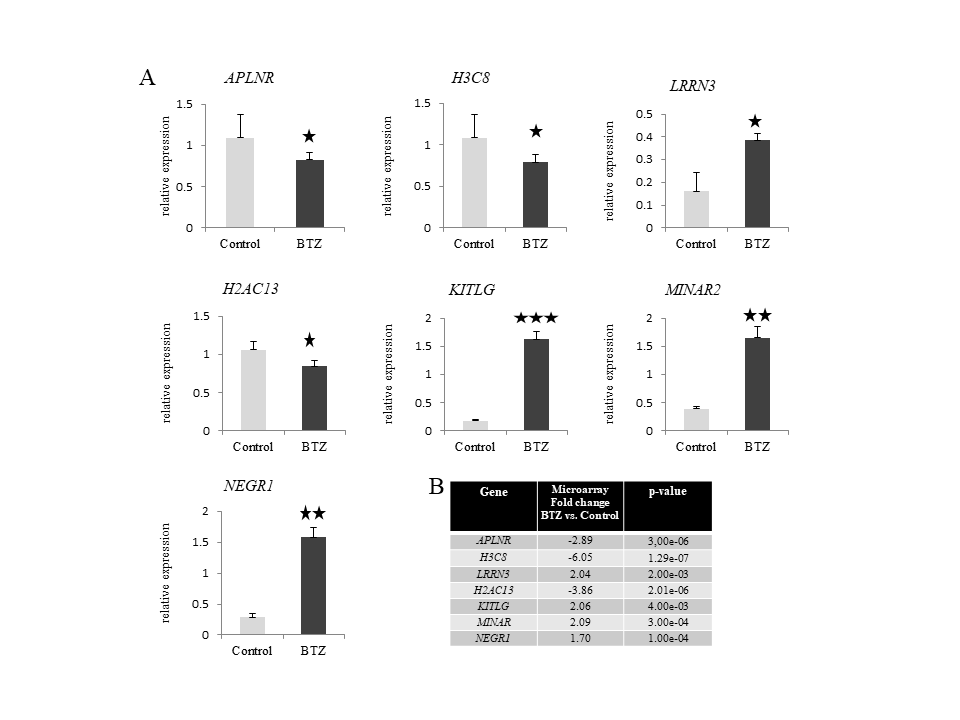

Supplement: Supplementary file 1 [file ijms-23-02431-s001.zip › fig. S1.tif]
